# A Maximum Likelihood Ensemble Filter via a Modified Cholesky Decomposition for Non-Gaussian Data Assimilation

**DOI:** 10.3390/s20030877

**Published:** 2020-02-06

**Authors:** Elias David Nino-Ruiz, Alfonso Mancilla-Herrera, Santiago Lopez-Restrepo, Olga Quintero-Montoya

**Affiliations:** 1 Applied Math and Computer Science Laboratory, Department of Computer Science, Universidad del Norte, Barranquilla 080001, Colombia; amancill@uninorte.edu.co; 2 Mathematical Modelling Research Group, Department of Mathematical Sciences, Universidad EAFIT, Medellín 050001, Colombia; slopezr2@eafit.edu.co (S.L.-R.); oquinte1@eafit.edu.co (O.Q.-M.); 3 Delft Institute of Applied Mathematics, Delft University of Technology, 2625 Delft, The Netherlands

**Keywords:** ensemble-based data assimilation, EnKF, MLEF, line-search optimization, modified cholesky decomposition, 49K10, 49M05, 49M15

## Abstract

This paper proposes an efficient and practical implementation of the Maximum Likelihood Ensemble Filter via a Modified Cholesky decomposition (MLEF-MC). The method works as follows: via an ensemble of model realizations, a well-conditioned and full-rank square-root approximation of the background error covariance matrix is obtained. This square-root approximation serves as a control space onto which analysis increments can be computed. These are calculated via Line-Search (LS) optimization. We theoretically prove the convergence of the MLEF-MC. Experimental simulations were performed using an Atmospheric General Circulation Model (AT-GCM) and a highly nonlinear observation operator. The results reveal that the proposed method can obtain posterior error estimates within reasonable accuracies in terms of ℓ−2 error norms. Furthermore, our analysis estimates are similar to those of the MLEF with large ensemble sizes and full observational networks.

## 1. Introduction

Remotely sensed observations by earth observing satellites are usually spatially and temporally discontinuous as a result of the sensor, satellite, and target view geometries [1]. For instance, polar orbiting satellites/sensors provide greater spatial details at a reduced temporal resolution, while geostationary orbiting satellites provide a better temporal resolution at a reduced spatial resolution [2]. Data Assimilation (DA) methods can be employed to make these observations more coherent both in time and space [3,4]. In this context, information from observations and an imperfect numerical forecast are optimally combined to estimate the state $x^{*}\in R^{n}$ of a dynamical system which approximately evolves according to some imperfect numerical model:

(1) $$\begin{array}{c} x_{\mathrm{next}}{=}_{M_{t_{\mathrm{current}}\to t_{\mathrm{next}}}}(x_{\mathrm{current}})\;,\;\mathrm{for}\;x\in R^{n}\;, \end{array}$$

where $M\;:\;R^{n}\to R^{n}$ is a numerical model which encapsulates our knowledge about the dynamic system of interest, *n* is the model resolution, and *t* stands for assimilation time. In sequential DA, well-known formulations are based on the cost function:

(2) $$\begin{array}{c} J(x)=\frac{1}{2}∥x-x^{b}{∥}_{B^{-1}}^{2}+\frac{1}{2}∥y-H(x){∥}_{R^{-1}}^{2}\;, \end{array}$$

where $x^{b}\in R^{n}$ is the background state, $B\in R^{n\times n}$ is the background error covariance matrix, $y\in R^{m}$ is a vector holding the observations, *m* is the number of observations, $R\in R^{m\times m}$ is the (estimated) data-error covariance matrix, and $H\;:\;R^{n}\to R^{m}$ is the observation operator (which maps model states to observations). Equation (2) is better known as the Three-Dimensional Variational (3D-Var) cost function. The analysis state is estimated via the solution of the 3D-Var optimization problem:

(3) $$\begin{array}{c} x^{a}=\arg\;\min_{x}\;J(x)\;, \end{array}$$

where $x^{a}\in R^{n}$ is the analysis state. For linear observation operators, closed-form solutions can be obtained for Equation (3), these are widely employed by ensemble-based methods. However, for nonlinear observation operators, numerical optimization methods can be employed to iteratively solve Equation (3). For instance, in the Maximum Likelihood Ensemble Filter (MLEF), vector states are constrained to the space spanned by an ensemble of model realizations, which is nothing but a low-rank square-root approximation of **B**. This method is widely accepted in the DA community owing to its efficient formulation and relative ease of implementation. Nevertheless, since analysis increments are computed onto an ensemble space, convergence is not ensured. We think that it is possible to replace the ensemble square-root approximation by a full-rank, well-conditioned square-root approximation of **B** via a modified Cholesky decomposition. In this manner, analysis increments are computed onto a space whose dimension equals that of the model one. Moreover, convergence can be ensured as long as the classic assumptions of Line-Search (LS) methods are satisfied.

The structure of this paper is as follows. In Section 2, we discuss ensemble-based methods for (non) linear data assimilation. Section 3.1 proposes a Maximum Likelihood Ensemble Filter via a Modified Cholesky decomposition (MLEF-MC); the theoretical aspects of this method as well as its computational cost are analyzed. In Section 4, numerical simulations are performed using the Lorenz-96 model and an Atmospheric General Circulation Model (AT-GCM). Section 5 states the conclusions of this research.

## 2. Preliminaries

In this section, we briefly discuss ensemble-based data assimilation in linear and nonlinear cases. Line-Search optimization methods are also discussed for the numerical solution of optimization problems.

### 2.1. Ensemble-Based Data Assimilation

Ensemble-based methods estimate prior error distributions via an ensemble of model realizations [5]:

(4) $$\begin{array}{c} X^{b}=[x^{b[1]},x^{b[2]},\ldots ,x^{b[N]}]\in R^{n\times N}\;, \end{array}$$

where *N* is the ensemble size, and $x^{b[e]}\in R^{n}$ stands for the *e*-th ensemble member, for 1≤e≤N. The empirical moments of Ensemble (4) are employed to estimate the moments of the prior error distributions:

(5) $$\begin{array}{c} x^{b}\approx {\overset{–}{x}}^{b}=\frac{1}{N}\sum_{e=1}^{N}x^{b[e]}\in R^{n}\;, \end{array}$$

and

(6) $$\begin{array}{c} B\approx P^{b}=\frac{1}{N}\Delta X\Delta X^{T}\in R^{n\times n}\;, \end{array}$$

where **ΔX** is the matrix of background anomalies:

(7) $$\begin{array}{c} \Delta X=X^{b}-{\overset{–}{x}}^{b}1^{T}\in R^{n\times N}\;, \end{array}$$

and **1** is a vector whose components are all ones. A well-known method in the ensemble context is the Ensemble Kalman Filter (EnKF) [6]. In the EnKF, a posterior ensemble can be built via the use of synthetic observation [7,8] or by employing an affine transformation on prior members [9,10]. Regardless which method is employed to estimate the analysis members, sampling errors impact the quality of the analysis members. This obeys the fact that, in practice, ensemble sizes are much smaller than model dimensions [11]. To counteract the effects of sampling noise, localization techniques are commonly employed during the assimilation steps. Localization relies on the idea that, for most geophysical systems, distant observations are weakly correlated [11,12]. Covariance localization and domain localization are frequently employed in operational scenarios. Furthermore, another possible choice is to make use of inverse covariance matrix estimation. In the EnKF based on a modified Cholesky decomposition [13,14,15], the precision covariance $B^{-1}$ is estimated via the Bickel and Levina covariance matrix estimator [16]. This estimator has the form

(8) $$\begin{array}{c} {\hat{B}}^{-1}={\hat{L}}^{T}{\hat{D}}^{-1}\hat{L}\in R^{n\times n}\;, \end{array}$$

where the nonzero components of $\hat{L}\in R^{n\times n}$ are obtained by fitting linear models of the form

(9) $$\begin{array}{c} x^{\left[i\right]}=\sum_{q\in \Pi (i,\;r)}{\left\{-\hat{L}\right\}}_{iq}^{}x^{\left[q\right]}+\eta^{\left[i\right]}\in R^{N}\;, \end{array}$$

where $x^{\left[i\right]}\in R^{N}$ is a vector holding the *i*-th model component across all ensemble members in Equation (7), for 1≤i≤n, and Π(i,r) denotes components within a local box of *i* for a radius size *r*. Note that the $\hat{L}$ factor is sparse since local neighborhoods are assumed for each model component. Moreover, it is possible to obtain sparse lower triangular factors by exploiting the mesh structures of numerical grids, that is, the sparsity pattern of $\hat{L}$ relies on the selection of *r*. Likewise, $\eta \in R^{N}$ is Gaussian with zero-mean and uncorrelated errors with unknown variance $\sigma^{2}$. Some structures of $\hat{L}$ are shown in Figure 1 for a one-dimensional grid and different values of *r*, cyclic boundary conditions are assumed for the physics/dynamics.

The ordering of model components plays an important role in an efficient manner to perform computations [17,18]. Thus, one can potentially exploit the special structure of the numerical mesh to obtain estimates which can be efficiently applied during the analysis steps [19]. However, the current literature proposes a modified Cholesky implementation, which can be applied without a prespecified ordering of model components [20].

EnKF methods commonly linearize observation operators when these are (highly) nonlinear [21], and as a direct consequence, this can induce bias on posterior members [22]. To handle nonlinear observation operators during the assimilation steps, optimization-based methods can be employed to estimate analysis increments. A well-known method in this context is the Maximum Likelihood Ensemble Filter (MLEF) [23]. This square-root filter employs the ensemble space to compute analysis increments [24,25]:

$$\begin{array}{c} {\overset{–}{x}}^{a}-{\overset{–}{x}}^{b}\in \mathrm{range}\{\Delta X\}\;, \end{array}$$

which is nothing but a pseudo square-root approximation of $B^{1/2}$. Thus, vector states can be written as follows:

(10) $$\begin{array}{c} x={\overset{–}{x}}^{b}+\Delta Xw\;, \end{array}$$

where $w\in R^{N}$ is a vector in redundant coordinates to be computed later. By replacing Equation (10) in Equation (2), one obtains [26,27]

(11) $$\begin{array}{c} J(x)=J({\overset{–}{x}}^{b}+\Delta Xw)=\frac{N-1}{2}{∥w∥}^{2}+\frac{1}{2}∥y-H({\overset{–}{x}}^{b}+\Delta Xw){∥}_{R^{-1}}^{2}\;. \end{array}$$

The optimization problem to solve reads

(12) $$\begin{array}{c} w^{*}=\arg\;\min_{w}\;J({\overset{–}{x}}^{b}+\Delta Xw)\;. \end{array}$$

This problem can be numerically solved via Line-Search (LS) and/or Trust-Region methods. However, convergence cannot be ensured as long as gradient approximations are performed onto a reduced space whose dimension is much smaller than that of the model.

### 2.2. Line-Search Optimization Methods

The solution of optimization problems of the form in Equation (3) can be approximated via Numerical Optimization [28,29]. In this context, solutions are obtained via iterations:

(13) $$\begin{array}{c} x_{k+1}=x_{k}+{\Delta x}_{k}\;, \end{array}$$

wherein *k* denotes the iteration index, and ${\Delta x}_{k}\in R^{n}$ is a descent direction, for instance, the gradient descent direction [30,31,32,33]

(14a) $$\begin{array}{c} {\Delta x}_{k}=-\nabla J(x_{k})\;, \end{array}$$

the Newton’s step [34,35,36],

(14b) $$\begin{array}{c} \nabla^{2}J(x_{k}){\Delta x}_{k}=-\nabla J(x_{k})\;, \end{array}$$

or a quasi-Newton-based method [37,38,39],

(14c) $$\begin{array}{c} P_{k}{\Delta x}_{k}=-\nabla J(x_{k})\;, \end{array}$$

where $P_{k}\in R^{n\times n}$ is a positive definite matrix. A concise survey of Newton-based methods can be consulted in [40]. Since step sizes in Equation (14) are based on first or second-order Taylor polynomials, the step size can be chosen via Line-Search [41,42,43] and/or Trust-Region [44,45,46] methods. Thus, we can ensure global convergence of optimization methods to stationary points of the cost function (2). This holds as long as some assumptions regarding the functions, gradients, and (potentially) Hessians are preserved [47]. In the context of Line-Search, the following assumptions are commonly made:

C-A A lower bound of J(x) exists on $\Omega_{0}=\{x\in R^{n},\;J(x)\leq J(x^{†})\}$, where $x^{†}\in R^{n}$ is available.
C-B There is a constant **L** such as
  $$\begin{array}{c} ∥\nabla J(x)-\nabla J(z)∥\leq L∥x-z∥,\;\;\mathrm{for}\;x,\;z\in B,\;\mathrm{and}\;L>0\;, \end{array}$$
  where *B* is an open convex set which contains $\Omega_{0}$. These conditions together with iterates of the form
  (15) $$\begin{array}{c} x_{k+1}=x_{k}+\alpha {\Delta x}_{k}\;, \end{array}$$
  ensure global convergence [48] as long as α is chosen as an (approximated) minimizer of
  (16) $$\begin{array}{c} \alpha^{*}=\arg\;\min_{\alpha \geq 0}\;J(x_{k}+\alpha {\Delta x}_{k})\;. \end{array}$$

In practice, rules for choosing step size such as the Goldstein rule [49], the Strong Wolfe rule [50], and the Halving method [51] are employed to partially solve Equation (16).

## 3. A Maximum Likelihood Ensemble Filter via a Modified Cholesky Decomposition

In this section, we develop an efficient and practical implementation of an MLEF-based filter via a modified Cholesky decomposition.

### 3.1. Filter Derivation

To solve the optimization problem (Equation (3)), we consider the matrix of anomalies (Equation (7)) to estimate $B^{-1}$ via a modified Cholesky decomposition. We then employ the square-root approximation

(17a) $$\begin{array}{c} {\hat{B}}^{1/2}=[{\hat{L}}^{T}{\hat{D}}^{1/2}{]}^{-1}\;, \end{array}$$

as a control space onto which analysis increments can be estimated. Note that

$$\begin{array}{c} \mathrm{rank}(B^{1/2})=\mathrm{rank}({\hat{B}}^{1/2})\;. \end{array}$$

We constrain vector states to the space spanned by Equation (17a):

(17b) $$\begin{array}{c} x={\overset{–}{x}}^{b}+{\hat{B}}^{1/2}\eta \;, \end{array}$$

where $\eta \in R^{n}$ is a vector of weights to be computed later. The 3D-Var cost function (Equation (2)) onto the space (Equation (17a)) reads

(17c) $$\begin{array}{c} J(x)=J({\overset{–}{x}}^{b}+{\hat{B}}^{1/2}\eta )=\frac{1}{2}{∥\eta ∥}^{2}+\frac{1}{2}∥y-H({\overset{–}{x}}^{b}+{\hat{B}}^{1/2}\eta ){∥}_{R^{-1}}^{2}\;, \end{array}$$

with the corresponding optimization problem:

(17d) $$\begin{array}{c} \eta^{*}=\arg\;\min_{\eta }\;J({\overset{–}{x}}^{b}+{\hat{B}}^{1/2}\eta )\;. \end{array}$$

To approximate a solution for Equation (17d), we consider iterates of the form

(18a) $$\begin{array}{c} x_{k+1}=x_{k}+{\hat{B}}^{1/2}\eta_{k}\;,\;\mathrm{for}\;0\leq k\leq K\;, \end{array}$$

with $x_{0}={\overset{–}{x}}^{b}$, where *k* denotes iteration index, and *K* is the maximum number of iterations. The weights $\eta_{k}$ can be computed as follows: at iteration *k*, we linearize the observation operator about $x_{k}$, this is

(18b) $$\begin{array}{c} H(x)\approx H(x_{k})+H_{k}[{\hat{B}}^{1/2}\eta_{k}]\;, \end{array}$$

where $H_{k}$ is the Jacobian of H(x) at $x_{k}$. By employing this linear Taylor expansion, we obtain the following quadratic approximation of Equation (17c):

(18c) $$\begin{array}{c} J(x_{k}+{\hat{B}}^{1/2}\eta_{k})\approx J_{k}(\eta_{k})=\frac{1}{2}∥\eta_{k}+\sum_{p=0}^{k-1}\eta_{p}{∥}^{2}+\frac{1}{2}∥\delta_{k}-{\hat{Q}}_{k}\eta_{k}{∥}_{R^{-1}}^{2}, \end{array}$$

where $\delta_{k}=y-H(x_{k})\in R^{m}$, and ${\hat{Q}}_{k}=H_{k}{\hat{B}}^{1/2}\in R^{m\times n}$. The gradient of Equation (18c) reads

(18d) $$\begin{array}{c} \nabla J_{k}(\eta_{k})=\eta_{k}+\sum_{p=0}^{k-1}\eta_{p}-{\hat{Q}}_{k}^{T}R^{-1}[\delta_{k}-{\hat{Q}}_{k}\eta_{k}]\;, \end{array}$$

from which an estimate of the optimal weight $\eta_{k}^{*}$ is as follows:

(18e) $$\begin{array}{c} \eta_{k}^{*}=[I+{\hat{Q}}_{k}^{T}R^{-1}{\hat{Q}}_{k}{]}^{-1}{\hat{Q}}_{k}^{T}R^{-1}\delta_{k}-\sum_{p=0}^{k-1}\eta_{p}^{*}. \end{array}$$

Since $\eta_{k}^{*}$ is obtained via a quadratic approximation of Equation (2), the step size (Equation (18e)) can be too large. Thus, we employ a Line-Search on Equation (17c) in the direction ${\hat{B}}^{1/2}\eta_{k}^{*}$:

(18f) $$\begin{array}{c} \rho_{k}^{*}=\arg\;\min_{\rho_{k}}J(x_{k}+\rho_{k}[{\hat{B}}^{1/2}\eta_{k}^{*}])\;, \end{array}$$

and therefore, by letting $\eta_{k}\approx \rho_{k}^{*}\eta_{k}^{*}$ in Equation (18a), we obtain

(18g) $$\begin{array}{c} x_{k+1}=x_{k}+{\hat{B}}^{1/2}[\rho_{k}^{*}\eta_{k}^{*}]\;. \end{array}$$

This process is repeated until a maximum number of iterations *K* is reached. Hence, an approximation of the optimal weight (Equation (17d)) reads

(19a) $$\begin{array}{c} \eta^{*}\approx \sum_{k=0}^{K}\eta_{k}\approx \sum_{k=0}^{K}\rho_{k}^{*}\eta_{k}^{*}\;, \end{array}$$

from which an estimate of the analysis state (Equation (3)) can be computed as follows:

(19b) $$\begin{array}{c} x^{a}\approx {\overset{–}{x}}^{a}={\overset{–}{x}}^{b}+{\hat{B}}^{1/2}[\sum_{k=0}^{K}\rho_{k}^{*}\eta_{k}^{*}]\;. \end{array}$$

The posterior covariance can be readily obtained from Equation (18d). Posterior weights can be sampled as follows:

(19c) $$\begin{array}{c} \eta^{a[e]}∼N(\eta^{*},\;[I+{\hat{Q}}_{K}^{T}R^{-1}{\hat{Q}}_{K}{]}^{-1})\;,\;\mathrm{for}\;1\leq e\leq N\;, \end{array}$$

and therefore, the analysis ensemble members read

(19d) $$\begin{array}{c} x^{a[e]}={\overset{–}{x}}^{b}+{\hat{B}}^{1/2}\eta^{a[e]}\;. \end{array}$$

The analysis members (Equation (19d)) are then propagated in time until new observations are available:

$$\begin{array}{c} x_{\mathrm{next}}^{b[e]}=M_{t_{\mathrm{current}}\to t_{\mathrm{next}}}(x_{\mathrm{next}}^{a[e]})\;. \end{array}$$

Putting it all together, the entire assimilation process is condensed in Algorithm 1. We call this filter formulation the Maximum Likelihood Ensemble Filter via a Modified Cholesky decomposition (MLEF-MC). Note that our goal is to obtain a minimizer (local optimum) of the 3D-Var optimization problem (Equation (3)). Other families of methods such as the Cluster Sampling Filters [52] target entire posterior density functions, that is, their goal is to draw samples from posterior kernels and using their empirical moments, to estimate posterior modes of error distributions.

### 3.2. Computational Cost of the MLEF-MC

We detail the computational cost of each line of Algorithm 1, and in this manner, we can estimate the overall computational cost of the MLEF-MC. We do not consider the computational cost of the Line-Search in Equation (18f), which will depend on the algorithm chosen for computing the optimal steps.

1. In Line 1, the direct inversion of matrix $B^{1/2}$ is not actually needed. Note that the optimization variable in Equation (17d) can be expressed in terms of a new control variable $\varsigma_{k}$ as follows:
  (20) $$\begin{array}{c} {}[{\hat{L}}^{T}{\hat{D}}^{-1/2}]\varsigma_{k}=\eta_{k}^{*}\;, \end{array}$$
  and in this manner, we can exploit the special structure of $\hat{L}$ and $\hat{D}$ to perform forward and backward substitutions on the optimal weights $\eta_{k}^{*}$. Thus, the number of computations to solve Equation (20) reads $O(\varphi^{2}n)$ where φ is the maximum number of nonzero entries across all rows in $\hat{L}$ with φ≪n. φ is commonly some function of the radius of influence *r*.
2. The computation of ${\hat{Q}}_{k}=H_{k}[{\hat{L}}^{T}{\hat{D}}^{-1/2}{]}^{-1}$ in Line 5 can be performed similarly to Equation (20). On the basis of the dimensions of $H_{k}$, a bound for computing ${\hat{Q}}_{k}$ is as follows: $O(\varphi^{2}nm)$.
3. In Line 7, the bounds for computations are as follows:
  $$\begin{array}{c} \eta_{k}^{*}=[I+{\hat{Q}}_{k}^{T}R^{-1}{\hat{Q}}_{k}{]}^{-1}\underset{O(nm)}{\underset{︸}{{\hat{Q}}_{k}^{T}\underset{O(m^{2})}{\underset{︸}{R^{-1}\delta_{k}}}}}-\sum_{p=0}^{k-1}\eta_{p}^{*}\;, \end{array}$$
  where the implicit linear system involving $I+{\hat{Q}}_{k}^{T}R^{-1}{\hat{Q}}_{k}$ can be solved, for instance, via the iterative Sherman Morrison formula [53] with no more than $O(\varphi^{2}nm)$ computations. Thus, the computational effort of computing Equation (18e) reads
  $$\begin{array}{c} O(\varphi^{2}nm+nm+m^{2})\;. \end{array}$$
  This bound is valid for Lines 9, 12, and 13. Since Lines 12 and 13 are performed *N* times, their computational cost reads $O(\varphi^{2}Nnm+nNm+{Nm}^{2})$. Since all computations are performed *K* times, the overall cost of the MLEF-MC is as follows:
  (21) $$\begin{array}{c} O(K[\varphi^{2}n+\varphi^{2}nm+\varphi^{2}nm+nm+m^{2}])\;. \end{array}$$

**Algorithm 1** Forecasts and Analysis Steps of the MLEF-MC
**Require:** Background ensemble members $\{x^{b[e]}{\}}_{e=1}^{N}$**Ensure:** An estimate of analysis members $\{x^{a[e]}{\}}_{e=1}^{N}$
1:Estimate ${\hat{B}}^{1/2}=[{\hat{L}}^{T}{\hat{D}}^{-1/2}{]}^{-1}$ via $\{x^{b[e]}{\}}_{e=1}^{N}$       ▹ Control space estimation2:Set $x_{0}\leftarrow {\overset{–}{x}}^{b}$                         ▹ Best estimation before observations3:**for**k←0→K**do**          ▹ Iterative solution of optimization problem (Equation (17d))4:    Compute the Jacobian $H_{k}$ of H(x) at $x_{k}$.5:    Set ${\hat{Q}}_{k}\leftarrow H_{k}{\hat{B}}^{1/2}$6:    Let $d_{k}\leftarrow y-H(x_{k})$7:    Compute:                                ▹*k*-th weight estimation
$$\begin{array}{c} \eta_{k}^{*}=[I+{\hat{Q}}_{k}^{T}R^{-1}{\hat{Q}}_{k}{]}^{-1}{\hat{Q}}_{k}^{T}R^{-1}\delta_{k}-\sum_{p=0}^{k-1}\delta_{p}^{*} \end{array}$$8:    Solve:                                ▹ Line-Search optimization
$$\begin{array}{c} \rho_{k}^{*}=\arg\;\min_{\rho_{k}}J(x_{k}+\rho_{k}[{\hat{B}}^{1/2}\eta_{k}^{*}])\;, \end{array}$$9:    Let $x_{k+1}\leftarrow x_{k}+{\hat{B}}^{1/2}[\rho_{k}^{*}\eta_{k}^{*}]$10:Set $\eta^{*}\leftarrow \sum_{k=0}^{K}\rho_{k}^{*}\eta_{k}^{*}$                    ▹Analysis weight11:**for**e←1→N**do**                        ▹ Analysis members computation12:    Set $\eta^{a[e]}∼N(\eta^{*},\;[I+{\hat{Q}}_{K}^{T}R^{-1}{\hat{Q}}_{K}{]}^{-1})$13:    Let $x^{a[e]}\leftarrow {\overset{–}{x}}^{b}+{\hat{B}}^{1/2}\eta^{a[e]}$14:**for**e←1→N**do**                                 ▹ Forecast step15:    Let $x_{\mathrm{next}}^{b[e]}\leftarrow M_{t_{\mathrm{current}}\to t_{\mathrm{next}}}(x_{\mathrm{current}}^{a[e]})$


### 3.3. Global Convergence of the Analysis Step in the MLEF-MC

To prove the global convergence of the proposed MLEF-MC in the analysis step, we consider the assumptions in Conditions (C-A), (C-B), and

(22) $$\begin{array}{c} \nabla J(x^{k}+{\hat{B}}_{k}\eta_{k}^{*}{)}^{T}{\hat{J}}_{k}(\eta_{k}^{*})<0,\;\;\mathrm{for}\;0\leq k\leq K\;. \end{array}$$

In the next theorem, we state the necessary conditions to ensure global convergence in the MLEF-MC method.

**Theorem** **1.** *If the Conditions (C-A), (C-B), and Equation (22) hold, then the MLEF-MC with exact Line-Search generates an infinite sequence $\{x_{k}{\}}_{k=0}^{\infty }$, then*

(23) $$\begin{array}{c} \lim_{k\to \infty }[\frac{-\nabla J(x_{k}{)}^{T}{\hat{B}}^{1/2}\eta_{k}^{*}}{∥{\hat{B}}^{1/2}\eta_{k}^{*}∥}{]}^{2}=0 \end{array}$$

*holds.*

**Proof.**  By Taylor series, the cost function (Equation (2)) can be expanded as follows:

$$\begin{array}{ccc} J(x_{k}+\rho_{k}^{*}{\hat{B}}^{1/2}\eta_{k}^{*}) & = & J(x_{k})\\ & + & \rho_{k}^{*}\int_{0}^{1}\;\nabla J(x_{k}+\rho_{k}^{*}t{\hat{B}}^{1/2}\eta_{k}^{*}{)}^{T}\\ & & {\hat{B}}^{1/2}\eta_{k}^{*}dt\;, \end{array}$$

and therefore,

$$\begin{array}{ccc} J(x_{k})-J\left(x_{k+1}\right) & \geq & -\rho_{k}^{*}\int_{0}^{1}\;\nabla J(x_{k}+\rho_{k}^{*}t{\hat{B}}^{1/2}\eta_{k}^{*}{)}^{T}\\ & & {\hat{B}}^{1/2}\eta_{k}^{*}dt \end{array}$$

for any $x_{k+1}$ on the ray $x_{k}+\rho_{k}{\hat{B}}^{1/2}\eta_{k}^{*}$, with ρ∈[0,1], we have

$$\begin{array}{c} J(x_{k})-J(x_{k+1})\geq J(x_{k})-J(x_{k}+\rho_{k}^{*}{\hat{B}}^{1/2}\eta_{k}^{*})\;, \end{array}$$

hence,

$$\begin{array}{ccc} J(x_{k}) & - & J(x_{k+1})\geq -\rho_{k}^{*}\nabla J(x_{k}{)}^{T}{\hat{B}}^{1/2}\eta_{k}^{*}\\ & - & \rho_{k}^{*}\int_{0}^{1}\;[\nabla J(x_{k}+\rho_{k}^{*}t{\hat{B}}^{1/2}\eta_{k}^{*})-\nabla J\left(x_{k}\right){]}^{T}\\ & & {\hat{B}}^{1/2}\eta_{k}^{*}dt\;, \end{array}$$

by the Cauchy–Schwarz inequality, we have

$$\begin{array}{ccc} J(x_{k}) & - & J(x_{k+1})\geq -\rho_{k}^{*}\nabla J(x_{k}{)}^{T}{\hat{B}}^{1/2}\eta_{k}^{*}\\ & - & \rho_{k}^{*}\int_{0}^{1}\;∥\nabla J(x_{k}+\rho_{k}^{*}t{\hat{B}}^{1/2}\eta_{k}^{*})-\nabla J(x_{k})∥\\ & & ∥{\hat{B}}^{1/2}\eta_{k}^{*}∥dt\\ & \geq & -\rho_{k}^{*}\nabla J(x_{k}{)}^{T}{\hat{B}}^{1/2}\eta_{k}^{*}\\ & - & \rho_{k}^{*}\int_{0}^{1}\;L∥\rho_{k}^{*}t{\hat{B}}^{1/2}\eta_{k}^{*}∥\;∥{\hat{B}}^{1/2}\eta_{k}^{*}∥dt\\ & = & -\rho_{k}^{*}\nabla J(x_{k}{)}^{T}{\hat{B}}^{1/2}\eta_{k}^{*}\\ & - & \rho_{k}^{*}L∥{\hat{B}}^{1/2}\eta_{k}^{*}∥\int_{0}^{1}∥t\rho_{k}^{*}{\hat{B}}^{1/2}\eta_{k}^{*}∥dt\\ & = & -\rho_{k}^{*}\nabla J(x_{k}{)}^{T}{\hat{B}}^{1/2}\eta_{k}^{*}-\frac{1}{2}\rho_{k}^{*2}L∥{\hat{B}}^{1/2}\eta_{k}^{*}{∥}^{2}\;, \end{array}$$

and choose

$$\begin{array}{c} \rho_{k}^{*}=-\frac{\nabla J(x_{k}{)}^{T}{\hat{B}}^{1/2}\eta_{k}^{*}}{L∥{\hat{B}}^{1/2}\eta_{k}^{*}{∥}^{2}}\;, \end{array}$$

therefore,

$$\begin{array}{ccc} J(x_{k}) & - & J(x_{k+1})\geq \frac{[\nabla J(x_{k}{)}^{T}{\hat{B}}^{1/2}\eta_{k}^{*}{]}^{2}}{L∥{\hat{B}}^{1/2}\eta_{k}^{*}{∥}^{2}}\\ & - & \frac{1}{2}\frac{[-\nabla J(x_{k}{)}^{T}{\hat{B}}^{1/2}\eta_{k}^{*}{]}^{2}}{L∥{\hat{B}}^{1/2}\eta_{k}^{*}{∥}^{2}}\\ & = & \frac{1}{2L}[-\frac{\nabla J(x_{k}{)}^{T}{\hat{B}}^{1/2}\eta_{k}^{*}}{∥{\hat{B}}^{1/2}\eta_{k}^{*}∥}{]}^{2}\;. \end{array}$$

By Condition (C-A), and Equation (22), it follows that $\{J(x_{k}){\}}_{k=0}^{\infty }$ is a monotone decreasing number sequence and it has a bound below, therefore $\{J(x_{k}){\}}_{k=0}^{\infty }$ has a limit, and consequently Equation (23) holds. □

### 3.4. Further Comments

Note that the proposed filter implementation performs the analysis step onto a control space whose dimension is equal to that of the model. This space is obtained via a modified Cholesky decomposition to mitigate the impact of sampling errors. Furthermore, its computational cost is linear with regard to the model size, which makes the MLEF-MC formulation for operational settings attractive. Moreover, the analysis step globally converges to posterior modes of the error distribution. The next section assesses the accuracy of our proposed filter implementation in several experimental settings.

## 4. Numerical Simulations

In this section, we test the proposed MLEF-MC implementation and compare our results with those obtained by the well-know MLEF method. We make use of two surrogate models for the experiments: the Lorenz-96 model [54] and an Atmospheric General Circulation Model (AT-GCM). In both cases, we consider the following general settings:

- Starting with a random solution, we employ the numerical model to obtain an initial condition which is consistent with the model dynamics. In a similar fashion, the background state, the actual state, and the initial ensemble are computed;
- We consider the following nonlinear observation operator [55]:
  (24) $$\begin{array}{c} {\left\{H(x)\right\}}_{j}=\frac{{\left\{x\right\}}_{j}}{2}\left[{\left(\frac{\left|{\left\{x\right\}}_{j}\right|}{2}\right)}^{\gamma -1}+1\right], \end{array}$$
  where *j* denotes the *j*-th observed component from the model state, for 1≤j≤m. Likewise, we vary γ in γ∈{1,3,5}. Note that we start with a linear observation operator and end up with a highly nonlinear one. Since this observation operator is nondifferentiable, we employ the sign function to approximate its derivative:
  $$\begin{array}{c} \frac{\partial H(x)}{\partial {\left\{x\right\}}_{j}}=\frac{{\left(\frac{\left|{\left\{x\right\}}_{j}\right|}{2}\right)}^{\gamma -1}}{2}+\frac{x\;\mathrm{sign}\left({\left\{x\right\}}_{j}\right)\;{\left(\frac{\left|{\left\{x\right\}}_{j}\right|}{2}\right)}^{\gamma -2}\;\left(\gamma -1\right)}{4}+\frac{1}{2}\;; \end{array}$$
- The ℓ−2 norm measures the accuracy of analysis states at assimilation stages,
  (25) $$\begin{array}{c} \lambda_{t}=\varepsilon (x_{\left[t\right]}^{*},\;x_{\left[t\right]}^{a})=\sqrt{{\left[x_{t}^{*}-x_{t}^{a}\right]}^{T}\left[x_{t}^{*}-x_{t}^{a}\right]}\;,\;\mathrm{for}\;0\leq t\leq M\;, \end{array}$$
  where $x_{t}^{*}$ and $x_{t}^{a}$ are the reference and the analysis solution at the assimilation step *t*, respectively;
- We employ the Root Mean Square Error (RMSE) as a measure of accuracy (average) for an entire set of time-spaced observations,
  (26) $$\begin{array}{c} \lambda =\sqrt{\frac{1}{M}\sum_{t=0}^{M}\lambda_{t}^{2}}\;; \end{array}$$
- We employ a Truncated Singular Value Decomposition (T-SVD) to fit the models (Equation (9));
- All experiments were performed under perfect model assumptions. No model errors were present during the assimilation steps;
- We employ the MLEF formulation proposed by Zupansky in [23].

### 4.1. The Lorenz-96 Model

**Figure 2:**
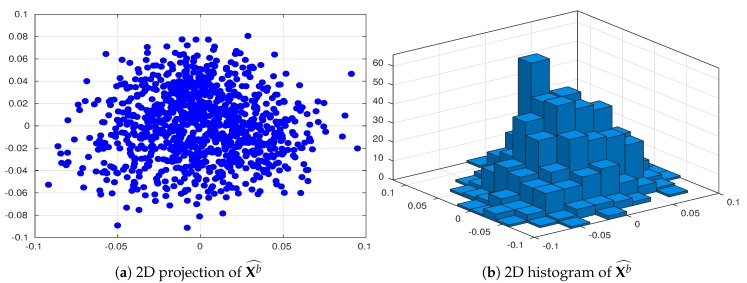
2D projections of the initial pool $\hat{X^{b}}$. Its two leading directions are employed for the projections.

The Lorenz-96 model is described by the following ordinary differential equations [56]:

(27) $$\begin{array}{c} \frac{dx_{j}}{dt}=\left\{\begin{array}{cc} \left(x_{2}-x_{n-1}\right)x_{n}-x_{1}+F & \mathrm{for}j=1,\\ \left(x_{j+1}-x_{j-2}\right)x_{j-1}-x_{j}+F & \mathrm{for}2\leq j\leq n-1,\\ \;\left(x_{1}-x_{n-2}\right)x_{n-1}-x_{n}+F & \mathrm{for}j=n, \end{array}\right. \end{array}$$

where *n* is the number of model variables, and *F* is the external force. Periodic boundary conditions are assumed. When F=8 and n=40, the model exhibits chaotic behavior, which makes it a relevant surrogate problem for atmospheric dynamics [57]. One time unit in the Lorenz-96 represents 7 days in the atmosphere. Details regarding the construction of the reference solution, background state, initial background ensemble member, and experimental settings are as follows:

- We create an initial pool $\hat{X^{b}}$ of $\hat{N}=1000$ ensemble members. For each experiment, we sample N=20 members from $\hat{X^{b}}$ to obtain the initial ensemble $X^{b}$. Two-dimensional projections of the initial pool making use of its two leading directions are shown in Figure 2;
- The assimilation window consists of M=100 time-spaced observations. Two observation frequencies are employed during the experiments: 16 h (time step of 0.1 time units) and 80 h (time step of 0.5 time units). We denote by δt∈{16,80} the time between two subsequent observations;
- At assimilation times, observational errors are characterized by Gaussian distributions with parameters
  $$\begin{array}{c} y∼N(H(x^{*}),\sigma_{o}^{2}I),\;\mathrm{for}\;0\leq t\leq M\;, \end{array}$$
  where $x^{*}$ is the actual state of the system, and $\sigma_{o}$ is the noise level. We tried three different noise levels for the observations $\sigma_{o}=\left\{0.01,\;0.1,\;1\right\}$;
- We consider two percentage of observations (*s*): 70% of model components (s=0.7) and 100% of model components (s=1). The components are randomly chosen at the different assimilation steps;
- The radii of influence to compute control spaces are ranged in r∈{1,3,5};
- The ensemble size for the MLEF-MC reads N=40;
- For a reference, we employ a MLEF method with an ensemble size of N=100 members and a full observational network s=1. Note that this ensemble size is more than twice the model resolution n=40. In this manner, we can have an idea about how errors should evolve for large ensemble sizes and full observational networks. We refer to this as the ideal case.

**Figure 3:**
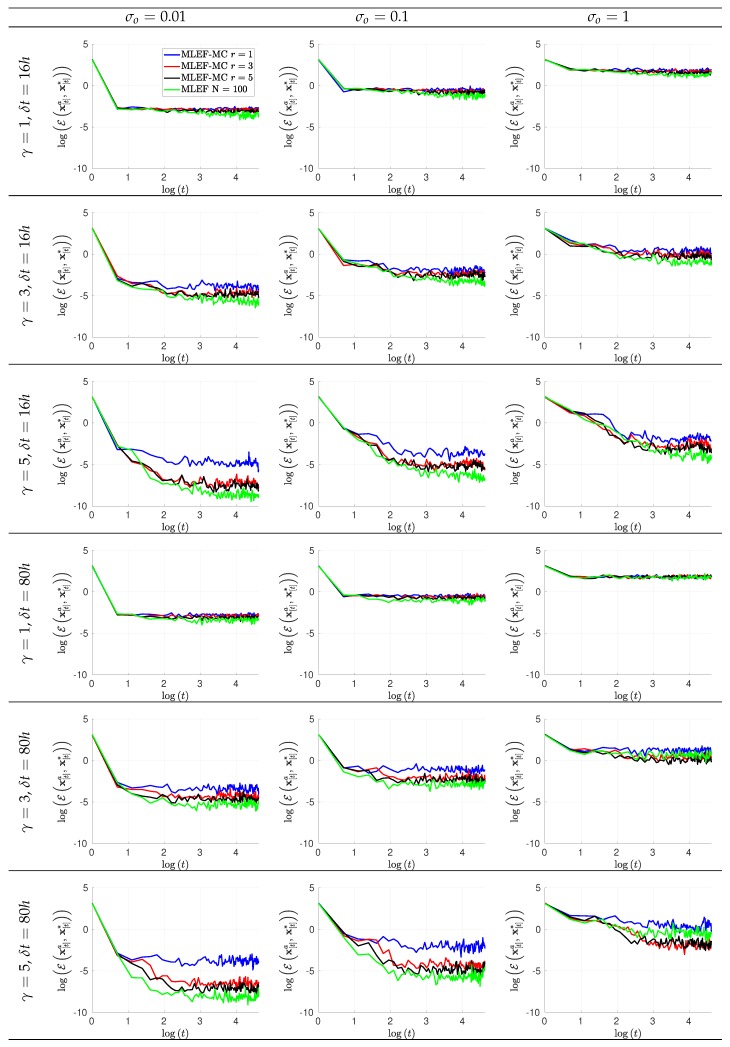
Error evolution in the log-scale of the compared filter implementations. Different time frequencies of observations were employed during the experiments. The percentage of observations from the model state reads s=100%.

**Figure 4:**
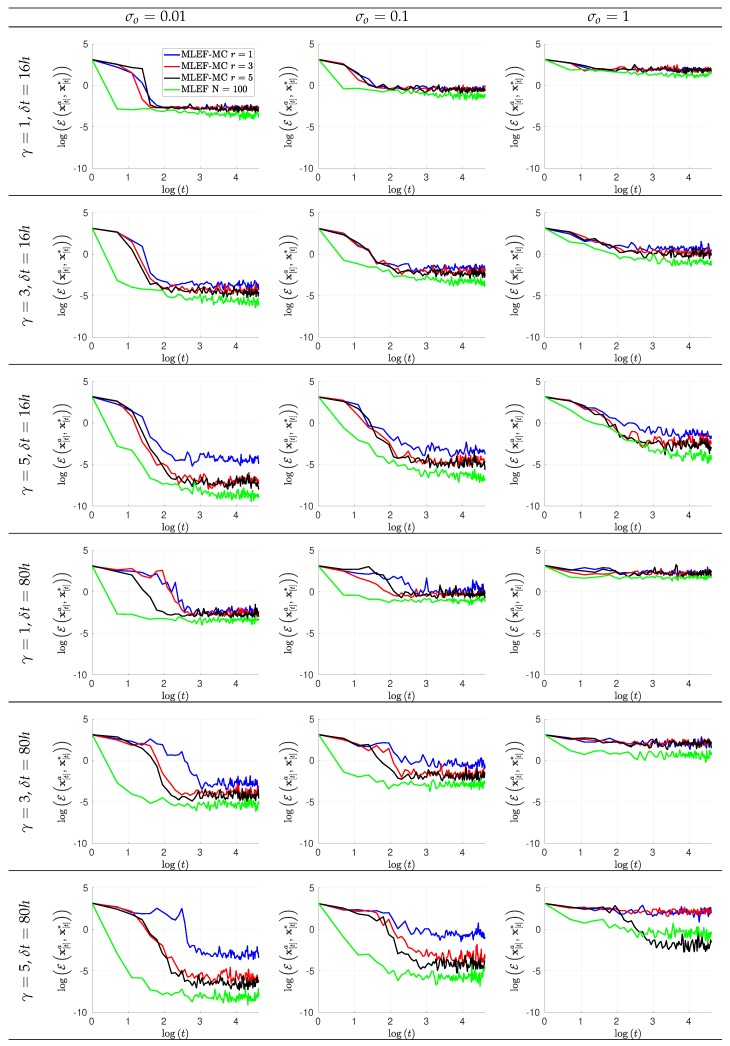
Error evolution in the log-scale of the compared filter implementations. Different time frequencies of observations were employed during the experiments. The percentage of observations from the model state reads s=70%.

The evolution of errors for the proposed filter implementation is detailed in Figure 3 and Figure 4 for the percentage of observations s=1 and s=0.7, respectively. We employ a log-scale of ℓ−2 error norms for ease of reading. Note that as the noise level $\sigma_{o}$ increases, the accuracy of the MLEF-MC degrades. This should be expected since more uncertainty is injected into the observations and as a direct consequence, the expected posterior errors increased. Nevertheless, in all cases, the evolution of errors are visually bounded (they do not blow up), and therefore, filter convergence is evidenced. For full observational networks, increments in the observation frequencies do not degrade the quality of the analysis increments; however, for observation coverages of s=0.7, the initial accuracies (spin-up period) can be impacted slightly as the observation frequency increases. However, this does not prevent errors from becoming stable (and to decrease) in time. Note that the degree γ of the observation operator does not impact the quality of analysis corrections in the MLEF-MC method. One can see that errors are stable in time regardless of the degree of H(x). On the other hand, the radius coverage plays an important role in the assimilation of observations as the time frequency of observations increases. For instance, as δt increases, the forecast steps are longer, and therefore, more information about background error correlations can be properly captured in our estimate ${\hat{B}}^{-1}$. Recall that background error correlations are driven by the nonlinear dynamics of the model (Equation (27)), and given the special structure of the ODE system (Equation (27)), it is reasonable to think that radius lengths larger than one can provide useful information to unobserved components during the analysis corrections. Thus, as the radius length increases, errors in the MLEF-MC behave similar to those in the ideal case.

**Figure 5:**
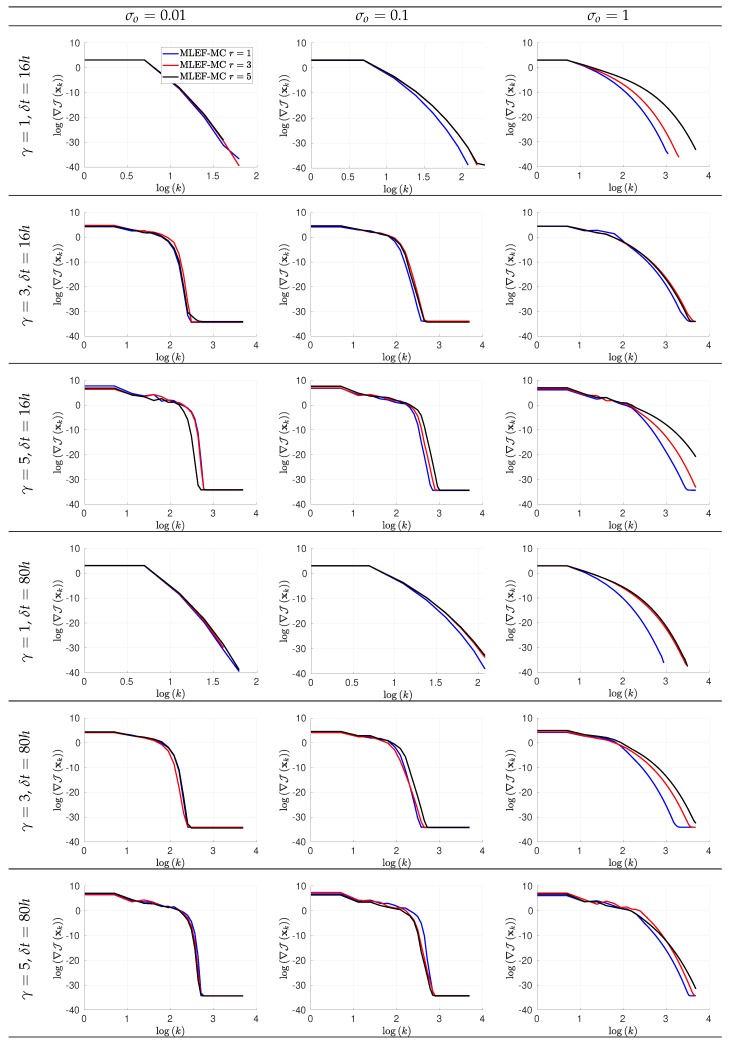
Gradient norms in the log-scale of the Maximum Likelihood Ensemble Filter via a Modified Cholesky decomposition (MLEF-MC) for the initial assimilation step. Different time frequencies of observations were employed during the experiments. The observation coverage from the model state reads s=100%.

**Figure 6:**
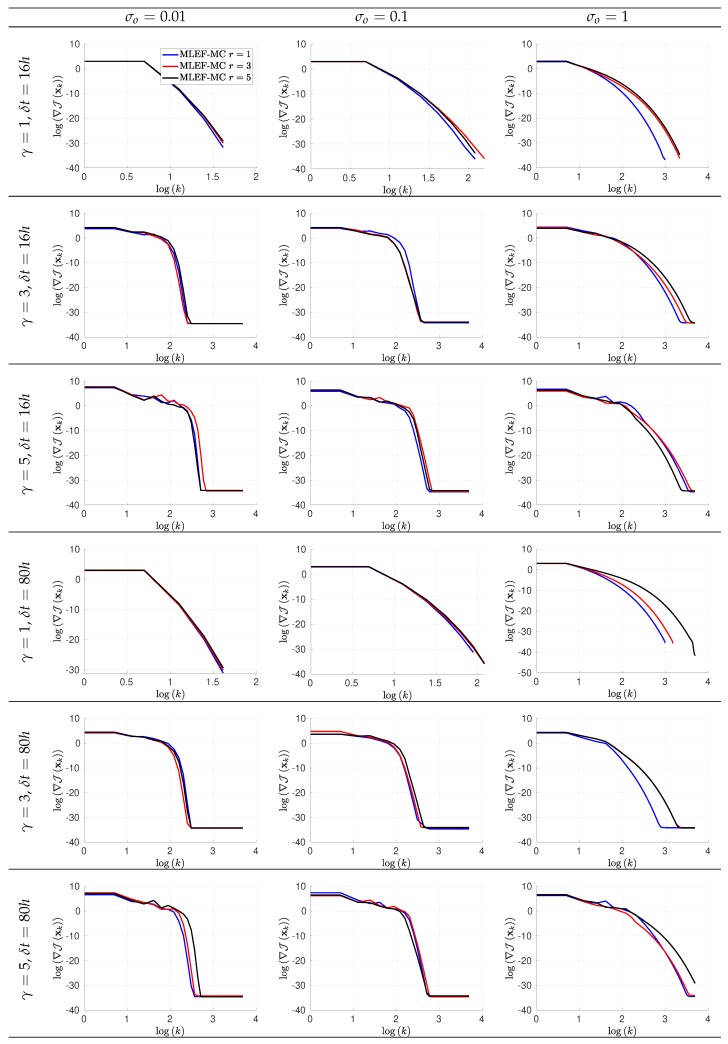
Gradient norms in the log-scale of the MLEF-MC for the initial assimilation step. Different time frequencies of observations were employed during the experiments. The observation coverage from the model state reads s=70%.

In Figure 5 and Figure 6, we report the gradient norms of the initial assimilation step for s=1 and s=0.7, respectively, for the MLEF-MC implementation. Note that for small γ and $\sigma_{o}$ values, gradient norms are similarly decreased for different values of *r* among iterations in the MLEF-MC context. As the noise level increases, high accuracies demand more iterations for large *r* values. Thus, the noise level plays an important role as long as radius lengths are increased. As should be expected, the rate of convergence can be impacted by the degree of the nonlinear observation operator. Recall that we employ a second-order approximation of J(x) to estimate its gradient, and therefore, as the degree γ increases, small step lengths will be employed by the Line-Search method, among iterations.

**Figure 7:**
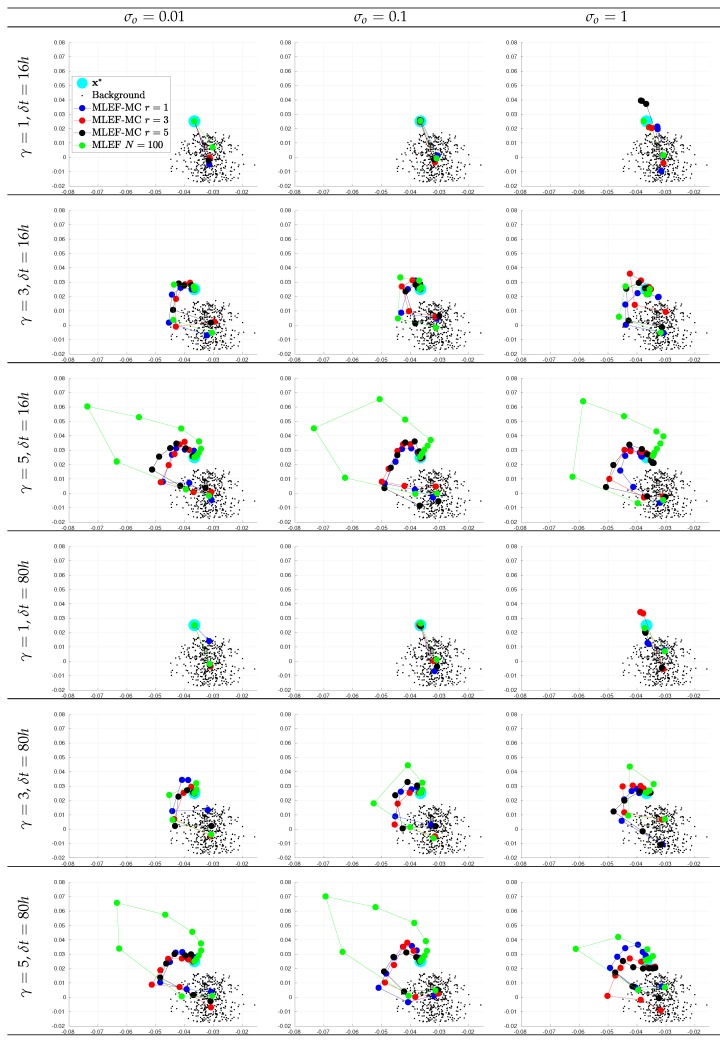
Snapshots of iterates for the initial analysis step. Different time frequencies of observations were employed during the experiments. The percentage of observations from the model state reads s=100%.

**Figure 8:**
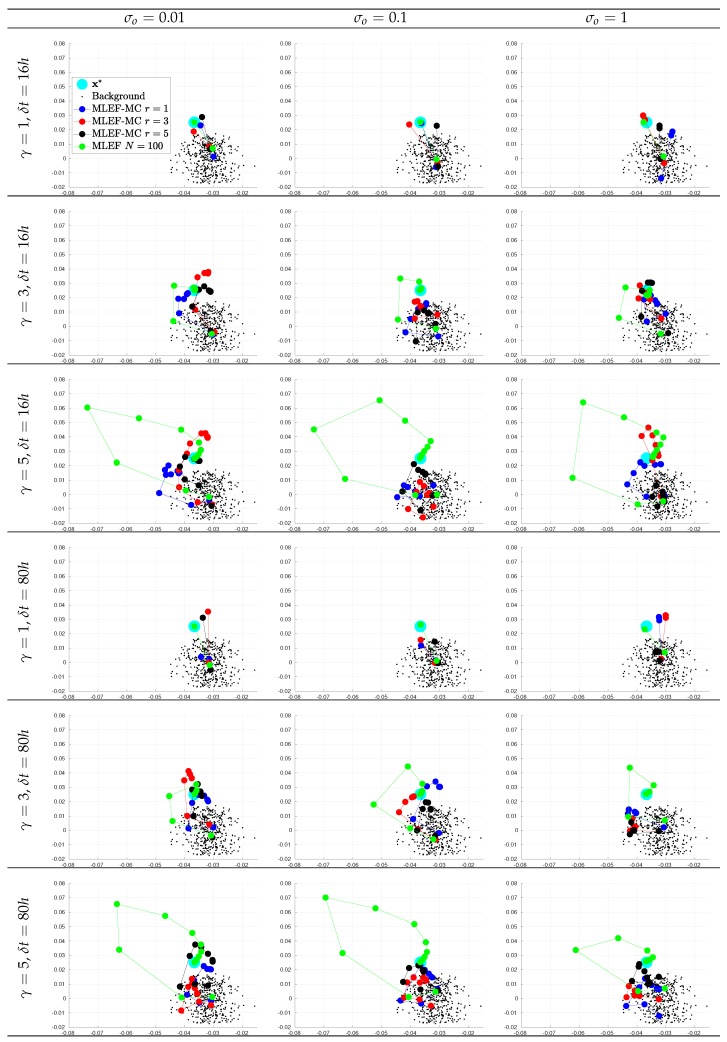
Snapshots of iterates for the initial analysis step. Different time frequencies of observations were employed during the experiments. The percentage of observations from the model state reads s=70%.

For the first assimilation cycle, we show two-dimensional projections of the optimization steps using the two leading directions of $\hat{X^{b}}$ in Figure 7 and Figure 8 for observation coverages of s=1 and s=0.7, respectively. We report the actual state $x^{*}$, some samples from the background error distribution $\hat{X^{b}}$, and the iterates for different *r* values. The ideal case is also reported. Note that as the degree γ increases, more iterations are needed before we obtain a reasonable estimate of $x^{*}$. As we mentioned before, second-order Taylor-based approximations can poorly estimate ∇J(x) as γ increases. As can be seen, as the noise level increases, the analysis estimate for the different radius lengths can be impacted.

**Figure 9:**
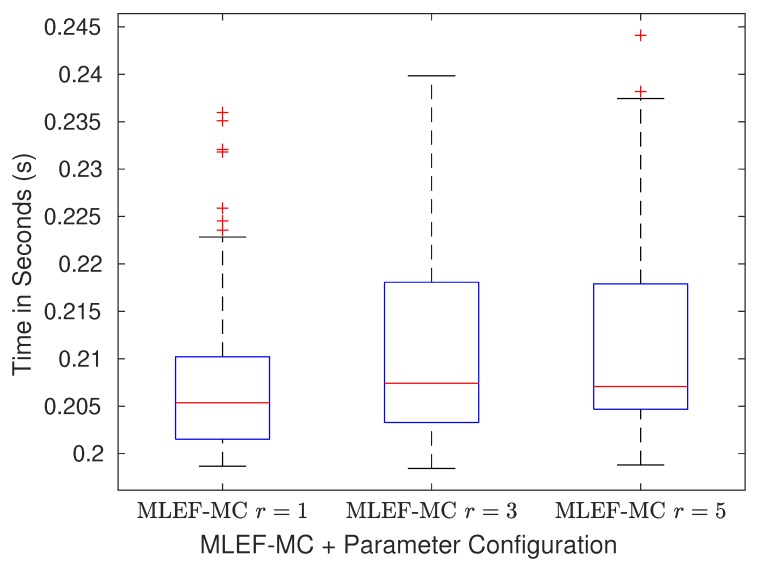
Average of elapsed times (in seconds) of the assimilation step for the MLEF-MC. Different parameters *r* were tried during experiments.

In Figure 9, we report the average of elapsed times for computing analysis increments across M=100 assimilation steps. As can be seen, as the radius of influence increases, the elapsed time of assimilation steps slightly increases. This agrees with the bound (Equation (21)) wherein the computational cost of the MLEF-MC formulation linearly depends on the model resolution *n*. Recall that the factor *r* is strictly related to φ, which in turn is bounded by *n*. In practice, φ≪n.

It is essential to note that by employing a modified Cholesky decomposition (Equation (8)), the degree of freedom of the control space (Equation (10)) is artificially increased. Thus, we have more directions (which are consistent with the model dynamics) onto which error dynamics can be captured. This is similar to having a localized square-root approximation of **B**. In this manner, we can decorrelate distant model components based on our prior knowledge about the model dynamics. Moreover, we can also decrease the impact of sampling errors. All these properties are possessed by our set of basis vectors (Equation (17a)), which can explain why our proposed filter implementation can decrease initial background errors by several orders of magnitudes. This obeys two important facts: the control-space dimension is equal to that of the model, and more importantly, MLEF-MC ensures convergence as long as the conditions of Theorem 1 are satisfied.

### 4.2. An Atmospheric General Circulation Model (AT-GCM)

In this section, we study the performance of the MLEF-MC method by using a highly nonlinear model: the SPEEDY model. This model is an atmospheric general circulation model that mimics the behavior of the atmosphere across different pressure levels [58,59]. The number of numerical layers in this model is seven, and we employ a T-30 spectral model resolution (96×48×7 grid components) for the space discretization of each model layer [60,61]. We employ four model variables. These are detailed in Table 1 with their corresponding units and the number of layers.

**Figure 10:**
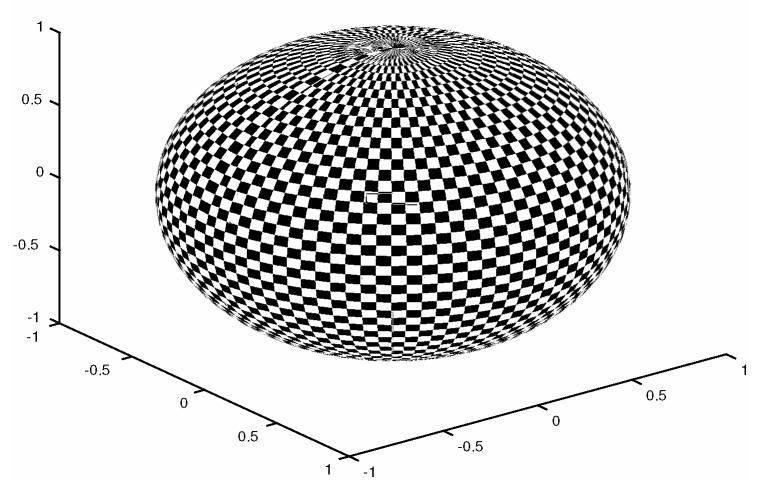
Linear observation operator during assimilation steps. Shaded regions denote observed components (observations) from the model state. The operator is replicated across all numerical layers.

Note that the total number of model components to be estimated is *n* = 133,632. We set the number of model realizations (ensemble size) as N=30 for all experimental scenarios. In this case, the model resolution is approximately 4454 times larger than the sample size (*n* ≫ *N*), which is very common under operational DA scenarios. Additional details of the experimental settings are described below, some are similar to those detailed in [62]:

- Starting with a system in equilibrium, the model is integrated over a long time period to obtain an initial condition whose dynamics are consistent with those of the SPEEDY model;
- The initial condition is perturbed *N* times and propagated over a long time period from which the initial background ensemble is obtained;
- We employ the trajectory of the initial condition as the reference. This reference trajectory serves to build synthetic observations;
- We set the standard deviations of errors in the observations as follows:
  - Temperature 1 K;
  - Zonal Wind Component 1 m/s;
  - Meridional Wind Component 1 m/s;
  - Specific Humidity $10^{-3}$ g/kg;
- Two percentages of observations are tried during the experiments: s=0.7 and s=1. Figure 10 shows an example of this operator;
- Observations are available every six hours (6 h);
- The experiments are performed under perfect model assumptions;
- The number of assimilation steps is M=12. Thus, the total simulation time is 7.5 days.

Table 2 shows the RMSE values for the MLEF-MC method. We vary the nonlinear degree of γ, and the percentage of observations *s*. Likewise, the radius of influence *r* is 1. As can be seen, the proposed filter implementation can decrease forecast errors for all model variables by, in some cases, several orders of magnitudes. As the degree of the observation operator increases, analysis errors can impact the analysis corrections, but all analysis errors are within the same orders of magnitude. Moreover, filter convergence is evident for all synthetic scenarios which agrees with Theorem 1. Note that as the number of observations increases, the accuracy of posterior estimates improves. This is expected since more information regarding the error dynamics is injected into the numerical forecast.

**Figure 11:**
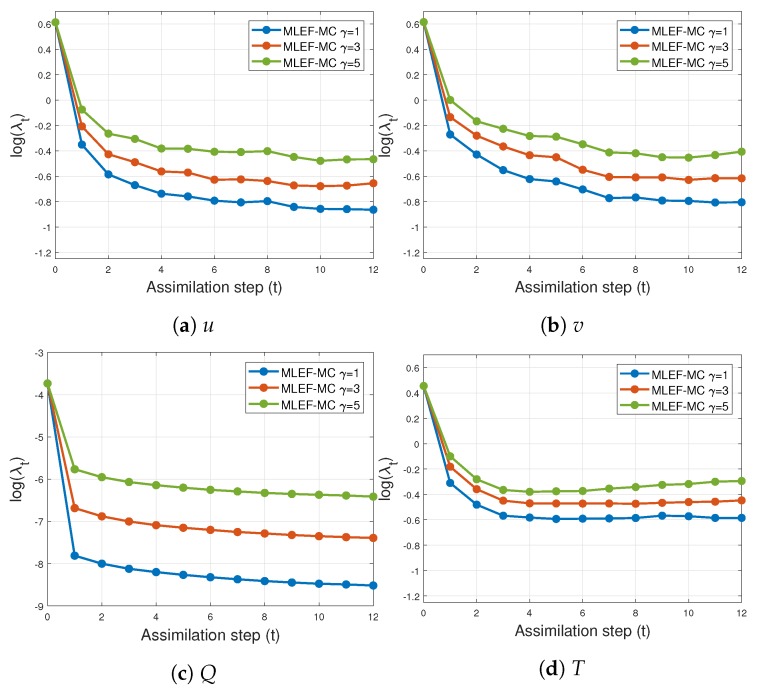
Time evolution of analysis errors for different values of parameters γ and *r* (MLEF-MC). s=1 (full observational network).

**Figure 12:**
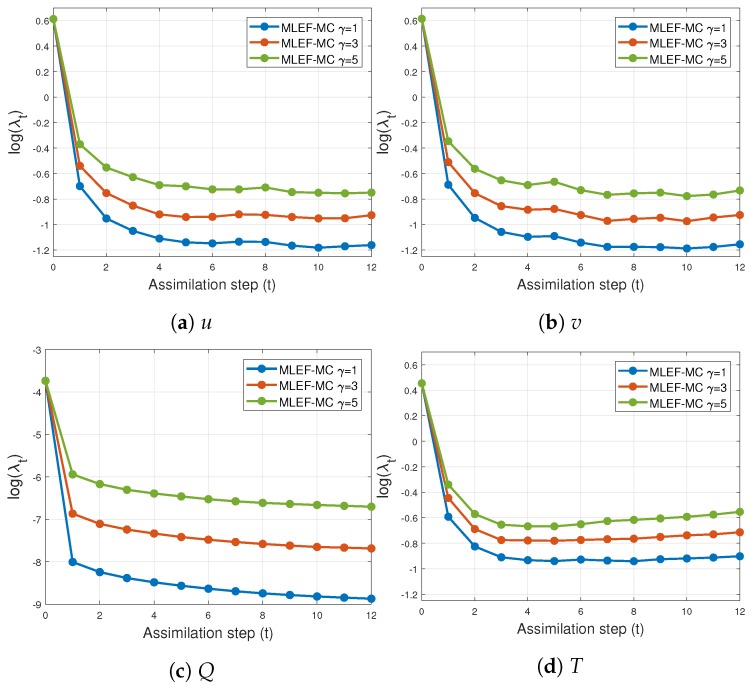
Time evolution of analysis errors for different values of parameters γ and *r* (MLEF-MC). s=1 (full observational network).

Figure 11 and Figure 12 show the time evolution of errors for s=0.7 and s=1.0, respectively. Clearly, initial errors are drastically decreased by the proposed filter implementation. This behavior is obtained regardless of the degree γ of the nonlinear observation operator (Equation (24)). As we mentioned before, the more observations employed during assimilation steps, the faster the posterior errors can be decreased. Furthermore, on the basis of the number of observations, the differences between posterior errors can be of orders of magnitude.

**Figure 13:**
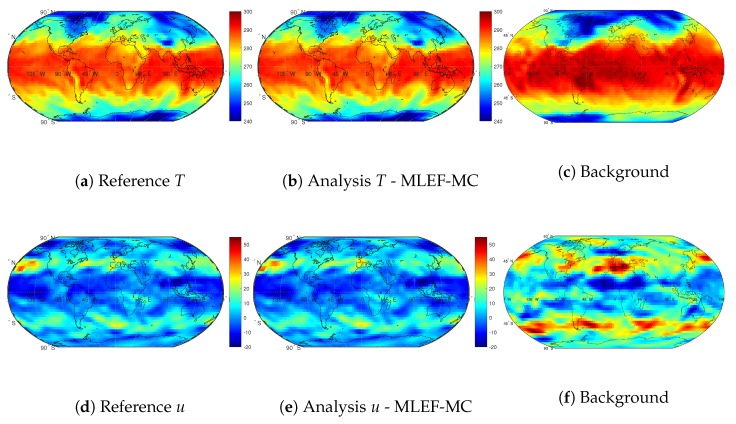
Snapshots of the SPEEDY model for the reference solution, the background estimate, and the analysis of the MLEF-MC. Results are shown for the first numerical layer (100 hPa), and the first assimilation step.

Figure 13 shows snapshots of the first assimilation step. The results are reported for the first numerical layer of the SPEEDY model and the model variables *u* and *T*. As can be seen, background errors are drastically improved by the MLEF-MC method. Spurious waves near the poles of the *T* and *u* variables are quickly dissipated, and the numerical model retains the actual shapes (and magnitudes) of these variables.

## 5. Conclusions

Satellite remote sensing with a wide range of sources, for instance, on-board sensors, platforms, and satellite data (which provides genuine earth observation information), has transformed our view of the Earth and its environment. These sensors offer different types of observations on large scales and over decades. Typically, observations (data) are nonlinearly related to model states. This paper proposes a Maximum Likelihood Ensemble Filter method via a Modified Cholesky decomposition (MLEF-MC) for nonlinear data assimilation. This method works as follows: snapshots of an ensemble of model realizations are taken at observation steps; these ensembles are employed to build control spaces onto which analysis increments can be estimated. The control spaces are obtained via a modified Cholesky decomposition. The control-space dimension is equal to that of the model, which mitigates the impact of sampling errors. Experimental tests were performed by using the Lorenz-96 model and an Atmospheric General Circulation Model (AT-GCM). The well-known Maximum Likelihood Ensemble Filter (MLEF) was employed with an ensemble size of 100 and a full observational network as a reference method to compare solutions. The results reveal that the proposed filter implementation performs similarly to the MLEF implementation (ideal case) in terms of $\ell_{2}$ error norms and Root Mean Square Error values.

## Figures and Tables

**Figure 1:**
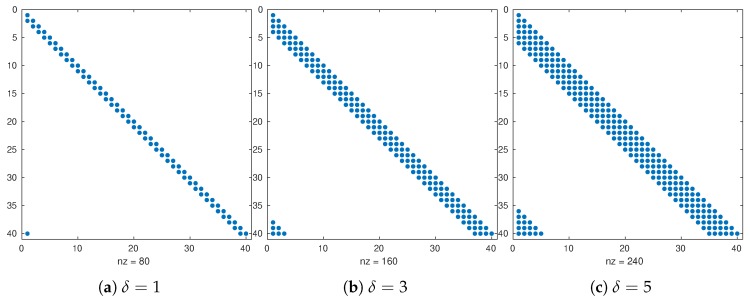
Structure of the Cholesky factor ${\hat{L}}_{k}$ as a function of the localization radius *r*.

**Table 1:** Physical variables of the SPEEDY model.

Name	Notation	Units	Number of Layers
Temperature	*T*	K	7
Zonal Wind Component	*u*	m/s	7
Meridional Wind Component	*v*	m/s	7
Specific Humidity	*Q*	g/kg	7

**Table 2:** Root Mean Square Error (RMSE) values for different values of *s* and γ.

Variable	NODA	*s* = 0.7			*s* = 1		
		γ=1	γ=3	γ=5	γ=1	γ=3	γ=5
*u* (m/s)	330.7048	0.6315	0.7113	0.7990	0.4703	0.5447	0.6232
*v* (m/s)	336.0850	0.5974	0.6742	0.7717	0.4708	0.5436	0.6266
*T* (K)	196.0983	0.6828	0.7416	0.8029	0.5402	0.6048	0.6629
*Q* (g/kg)	0.1010	0.0032	0.0070	0.0135	0.0026	0.0058	0.0113

## References

[1] Asner G.P., Warner A.S. (2003). Canopy shadow in IKONOS satellite observations of tropical forests and savannas. Remote Sens. Environ..

[2] Mayr S., Kuenzer C., Gessner U., Klein I., Rutzinger M. (2019). Validation of Earth Observation Time-Series: A Review for Large-Area and Temporally Dense Land Surface Products. Remote Sens..

[3] Jin X., Kumar L., Li Z., Feng H., Xu X., Yang G., Wang J. (2018). A review of data assimilation of remote sensing and crop models. Eur. J. Agron..

[4] Khaki M. (2020). Data Assimilation and Remote Sensing Data. Satellite Remote Sensing in Hydrological Data Assimilation.

[5] Evensen G. (2003). The Ensemble Kalman Filter: Theoretical formulation and practical implementation. Ocean Dyn..

[6] Evensen G. (1994). Sequential data assimilation with a nonlinear quasi-geostrophic model using Monte Carlo methods to forecast error statistics. J. Geophys. Res. Oceans.

[7] Nino-Ruiz E.D., Sandu A. (2015). Ensemble Kalman filter implementations based on shrinkage covariance matrix estimation. Ocean Dyn..

[8] Nino-Ruiz E.D., Sandu A., Deng X. (2018). An Ensemble Kalman Filter Implementation Based on Modified Cholesky Decomposition for Inverse Covariance Matrix Estimation. SIAM J. Sci. Comput..

[9] Bishop C.H., Etherton B.J., Majumdar S.J. (2001). Adaptive sampling with the ensemble transform Kalman filter. Part I: Theoretical aspects. Mon. Weather Rev..

[10] Hunt B.R., Kostelich E.J., Szunyogh I. (2007). Efficient data assimilation for spatiotemporal chaos: A local ensemble transform Kalman filter. Physica D.

[11] Petrie R.E. (2008). Localization in the Ensemble Kalman Filter. Master’s Thesis.

[12] Hamill T.M., Whitaker J.S., Snyder C. (2001). Distance-Dependent Filtering of Background Error Covariance Estimates in an Ensemble Kalman Filter. Mon. Weather Rev..

[13] Nino-Ruiz E.D., Sandu A., Deng X. A parallel ensemble Kalman filter implementation based on modified Cholesky decomposition. Proceedings of the 6th Workshop on Latest Advances in Scalable Algorithms for Large-Scale Systems.

[14] Nino-Ruiz E.D., Sandu A., Deng X. (2019). A parallel implementation of the ensemble Kalman filter based on modified Cholesky decomposition. J. Comput. Sci..

[15] Nino-Ruiz E. (2017). A matrix-free posterior ensemble kalman filter implementation based on a modified cholesky decomposition. Atmosphere.

[16] Bickel P.J., Levina E. (2008). Regularized estimation of large covariance matrices. Ann. Stat..

[17] Dellaportas P., Pourahmadi M. (2012). Cholesky-GARCH models with applications to finance. Stat. Comput..

[18] Rajaratnam B., Salzman J. (2013). Best permutation analysis. J. Multivar. Anal..

[19] Kang X., Deng X., Tsui K.W., Pourahmadi M. (2019). On variable ordination of modified Cholesky decomposition for estimating time-varying covariance matrices. Int. Stat. Rev..

[20] Zheng H., Tsui K.W., Kang X., Deng X. (2017). Cholesky-based model averaging for covariance matrix estimation. Stat. Theor. Relat. Fields.

[21] Bertino L., Evensen G., Wackernagel H. (2007). Sequential Data Assimilation Techniques in Oceanography. Int. Stat. Rev..

[22] Zupanski M., Navon I.M., Zupanski D. (2008). The Maximum Likelihood Ensemble Filter as a non-differentiable minimization algorithm. Q. J. R. Meteorol. Soc..

[23] Zupanski M. (2005). Maximum Likelihood Ensemble Filter: Theoretical Aspects. Mon. Weather Rev..

[24] Fletcher S.J., Zupanski M. (2008). A study of ensemble size and shallow water dynamics with the Maximum Likelihood Ensemble Filter. Tellus A.

[25] Carrassi A., Vannitsem S., Zupanski D., Zupanski M. (2009). The maximum likelihood ensemble filter performances in chaotic systems. Tellus A.

[26] Tran A.P., Vanclooster M., Zupanski M., Lambot S. (2014). Joint estimation of soil moisture profile and hydraulic parameters by ground-penetrating radar data assimilation with maximum likelihood ensemble filter. Water Resour. Res..

[27] Zupanski D., Zupanski M. (2006). Model Error Estimation Employing an Ensemble Data Assimilation Approach. Mon. Weather Rev..

[28] Vanderplaats G.N. (1984). Numerical Optimization Techniques for Engineering Design: With Applications.

[29] Wright S., Nocedal J. (1999). Numerical optimization.

[30] Savard G., Gauvin J. (1994). The steepest descent direction for the nonlinear bilevel programming problem. Oper. Res. Lett..

[31] Hager W.W., Zhang H. (2006). A survey of nonlinear conjugate gradient methods. Pac. J. Optim..

[32] Fletcher R., Reeves C.M. (1964). Function minimization by conjugate gradients. Comput. J..

[33] Lewis R.M., Torczon V., Trosset M.W. (2000). Direct search methods: Then and now. J. Comput. Appl. Math..

[34] Battiti R. (1992). First-and second-order methods for learning: Between steepest descent and Newton’s method. Neural Comput..

[35] Grippo L., Lampariello F., Lucidi S. (1989). A truncated Newton method with nonmonotone line search for unconstrained optimization. J. Optim. Theory Appl..

[36] Pan V.Y., Branham S., Rosholt R.E., Zheng A.L. (1999). Newton’s iteration for structured matrices. Fast Reliable Algorithms for Matrices with Structure.

[37] Shanno D.F. (1970). Conditioning of quasi-Newton methods for function minimization. Math. Comput..

[38] Nocedal J. (1980). Updating quasi-Newton matrices with limited storage. Math. Comput..

[39] Loke M.H., Barker R. (1996). Rapid least-squares inversion of apparent resistivity pseudosections by a quasi-Newton method. Geophys. Prospect..

[40] Knoll D.A., Keyes D.E. (2004). Jacobian-free Newton–Krylov methods: A survey of approaches and applications. J. Comput. Phys..

[41] Grippo L., Lampariello F., Lucidi S. (1986). A nonmonotone line search technique for Newton’s method. SIAM J. Numer. Anal..

[42] Uschmajew A., Vandereycken B. Line-search methods and rank increase on low-rank matrix varieties. Proceedings of the 2014 International Symposium on Nonlinear Theory and Its Applications (NOLTA2014).

[43] Hosseini S., Huang W., Yousefpour R. (2018). Line search algorithms for locally Lipschitz functions on Riemannian manifolds. SIAM J. Optim..

[44] Conn A.R., Gould N.I., Toint P.L. (2000). Trust Region Methods.

[45] Moré J.J., Sorensen D.C. (1983). Computing a trust region step. SIAM J. Sci. Comput..

[46] Curtis F.E., Robinson D.P., Samadi M. (2017). A trust region algorithm with a worst-case iteration complexity of O(*ϵ*^−3/2^) for nonconvex optimization. Math. Program..

[47] Shi Z.J. (2004). Convergence of line search methods for unconstrained optimization. Appl. Math. Comput..

[48] Zhou W., Akrotirianakis I., Yektamaram S., Griffin J. (2017). A matrix-free line-search algorithm for nonconvex optimization. Optim. Methods Softw..

[49] Dunn J.C. (1980). Newton’s method and the Goldstein step-length rule for constrained minimization problems. SIAM J. Control Optim..

[50] Dai Y.H., Yuan Y. (1999). A nonlinear conjugate gradient method with a strong global convergence property. SIAM J. Optim..

[51] Ravindran A., Reklaitis G.V., Ragsdell K.M. (2006). Engineering Optimization: Methods and Applications.

[52] Attia A., Moosavi A., Sandu A. (2018). Cluster sampling filters for non-Gaussian data assimilation. Atmosphere.

[53] Nino-Ruiz E.D., Sandu A., Anderson J. (2015). An efficient implementation of the ensemble Kalman filter based on an iterative Sherman–Morrison formula. Stat. Comput..

[54] Lorenz E.N. (2005). Designing Chaotic Models. J. Atmos. Sci..

[55] Van Leeuwen P.J. (2010). Nonlinear data assimilation in geosciences: An extremely efficient particle filter. Q. J. R. Meteorol. Soc..

[56] Gottwald G.A., Melbourne I. (2005). Testing for chaos in deterministic systems with noise. Physica D.

[57] Karimi A., Paul M. (2010). Extensive Chaos in the Lorenz-96 Model. Chaos.

[58] Bracco A., Kucharski F., Kallummal R., Molteni F. (2004). Internal variability, external forcing and climate trends in multi-decadal AGCM ensembles. Clim. Dyn..

[59] Miyoshi T. (2011). The Gaussian approach to adaptive covariance inflation and its implementation with the local ensemble transform Kalman filter. Mon. Weather Rev..

[60] Molteni F. (2003). Atmospheric simulations using a GCM with simplified physical parametrizations. I: Model climatology and variability in multi-decadal experiments. Clim. Dyn..

[61] Kucharski F., Molteni F., Bracco A. (2006). Decadal interactions between the western tropical Pacific and the North Atlantic Oscillation. Clim. Dyn..

[62] Miyoshi T., Kondo K., Imamura T. (2014). The 10,240-member ensemble Kalman filtering with an intermediate AGCM. Geophys. Res. Lett..

